# Semantic Web repositories for genomics data using the eXframe platform

**DOI:** 10.1186/2041-1480-5-S1-S3

**Published:** 2014-06-03

**Authors:** Emily Merrill, Stéphane Corlosquet, Paolo Ciccarese, Tim Clark, Sudeshna Das

**Affiliations:** 1 Massachusetts General Hospital, Partners Research Building, 65 Landsdowne St, Cambridge, MA, 02139, USA; 2 Harvard Medical School, 25 Shattuck St, Boston, MA, 02115, USA; 3 School of Computer Science, University of Manchester, Oxford Road, Manchester, M13 9PL, UK

## Abstract

**Background:**

With the advent of inexpensive assay technologies, there has been an unprecedented growth in genomics data as well as the number of databases in which it is stored. In these databases, sample annotation using ontologies and controlled vocabularies is becoming more common. However, the annotation is rarely available as Linked Data, in a machine-readable format, or for standardized queries using SPARQL. This makes large-scale reuse, or integration with other knowledge bases very difficult.

**Methods:**

To address this challenge, we have developed the second generation of our eXframe platform, a reusable framework for creating online repositories of genomics experiments. This second generation model now publishes Semantic Web data. To accomplish this, we created an experiment model that covers provenance, citations, external links, assays, biomaterials used in the experiment, and the data collected during the process. The elements of our model are mapped to classes and properties from various established biomedical ontologies. Resource Description Framework (RDF) data is automatically produced using these mappings and indexed in an RDF store with a built-in Sparql Protocol and RDF Query Language (SPARQL) endpoint.

**Conclusions:**

Using the open-source eXframe software, institutions and laboratories can create Semantic Web repositories of their experiments, integrate it with heterogeneous resources and make it interoperable with the vast Semantic Web of biomedical knowledge.

## Background

There has been a rapid cost reduction per megabase of genomic information obtained, beating Moore's law [1] many-fold [2,3], resulting in an exponential growth of genomics data, especially next generation sequencing data [4]. Standards to unambiguously describe the experimental details are required to facilitate the understanding, quality checking, reusing, reproducing and integrating the data. The bioinformatics community has responded to the challenge and several standards have been developed over the years. The first standard to be published provided requirements for the Minimum Information About a Microarray Experiment (MIAME) [5]. Several other standards were published as new technologies evolved and then the Minimum Information for Biological and Biomedical Investigations guideline was proposed for reporting all types of biomedical experiments [6]. The major public repositories of genomics experiments, Gene Expression Omnibus (GEO) [7] and ArrayExpress [8], are compliant with these standards.

While standards addressed the need for uniform experiment representation, controlled vocabularies, terminologies and ontologies were developed to describe the samples, assays and other experimental details in an unambiguous manner. For example, the Ontology for Biomedical Investigations (OBI) [9] provides a model for biomedical experiments with classes that describe elements of the experimental investigation process. The Experimental Factor Ontology (EFO) [10] was developed as an application ontology to describe the genomics data in ArrayExpress [8]. In addition several ontologies and vocabularies have also been developed to describe biological specimens such as the organism, tissue, cell type, disease state. These include the Cell Ontology (CL) [11], the Foundation Model of Anatomy (FMA) [12], Disease Ontology (DO) [13] among numerous others.

Several repositories of genomics data have adopted the MIAME or MIBBI standards and are leveraging these biomedical ontologies to provide consistent annotation of experiments. A few examples from diverse domains include the Gemma repository - a resource for sharing, reuse and meta-analysis of microarray data [14], Chemical Effects in Biological Systems (CEBS) database that contains data of interest to environmental health scientists [15] and Oncomine an integrated database and mining platform for oncology data mine [16]. Although these resources make use of ontologies to represent experimental data in a standardized manner, the annotations are not machine-readable by other software and thus integration with other knowledge resources remain a challenge.

Meanwhile, Semantic Web [17] technologies such as Linked Data, Resource Description Framework (RDF) and SPARQL are increasingly being used in the bioinformatics community to respond to the knowledge integration needs [18]. Semantic Web allows one to query across disparate resources using a single flexible interface. For example, the Bio2RDF project successfully applies Semantic Web technologies to create a mashup of key publicly available databases using a common ontology and normalized Uniform Resource Identifiers (URI) [19,20]. Cheung et al. demonstrate the use of Semantic Web technologies for a federated query in the neuroscience domain [21]. There are several other examples across various biomedical domains that demonstrate the power of Semantic Web technologies.

However, surprisingly there has been no wide spread adoption of Semantic Web technologies for experiment repositories, where queries using domain ontologies can help bridge different disciplines, for important applications such as translational medicine. Recently the European Bioinformatics Institute (EBI), recognizing this urgent need, has released an RDF platform that includes a SPARQL endpoint for the Gene Expression Atlas [22], a database that summarizes gene expression from ArrayExpress experiments[23]. However, it doesn't provide reusable software that can be used by other institutions to house and query their genomics data.

To address this gap, we developed eXframe as a reusable software platform to build genomics repositories that automatically produce Linked Data and a SPARQL endpoint. Our platform is based on an open source content management system and uses existing biomedical ontologies to produce Semantic Web data enabling interoperability with the other resources. The code is freely available and application is demonstrated with a repository of stem cell data.

## Implementation

In this section we describe the implementation of eXframe and how it automatically generates Linked Data.

### Framework

The eXframe software framework [24] enables creation of web-based genomics experiment repositories. It is based on an open source content management system, Drupal [25], with modifications to support genomic experiment data. In this paper, we report a re-factored second generation of eXframe, which produces Linked Data and a SPARQL endpoint for querying it. The revised version also includes an updated experiment model that has been generalized to support various types of biomedical experiments as well as an upgrade to Drupal 7.

**Figure 1:**
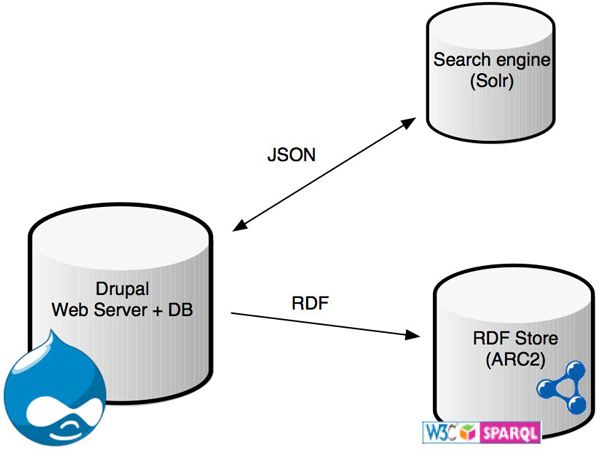
**eXframe architecture**. Overall schematic of eXframe architecture displaying the major components.

We have defined content types (e.g. experiments, assays, biomaterials and bibliographic citations) as well as their relationships as first class objects in Drupal. These predefined content types are packaged as Drupal features and available for use within eXframe. All content types and their fields are mapped to appropriate ontologies and vocabularies as described in the following section. Using these mappings, the Drupal RDF modules [26] are used to produce RDF as well as a SPARQL endpoint. Data can also be exported in other standard formats such as ISA-Tab [27]. A simple schematic of the architecture is shown in Figure 1. The software also includes a basic theme (colors, fonts and style) for the website. Any group or institution that uses eXframe can customize the content types, theme or ontology mappings.

### Data model

**Figure 2:**
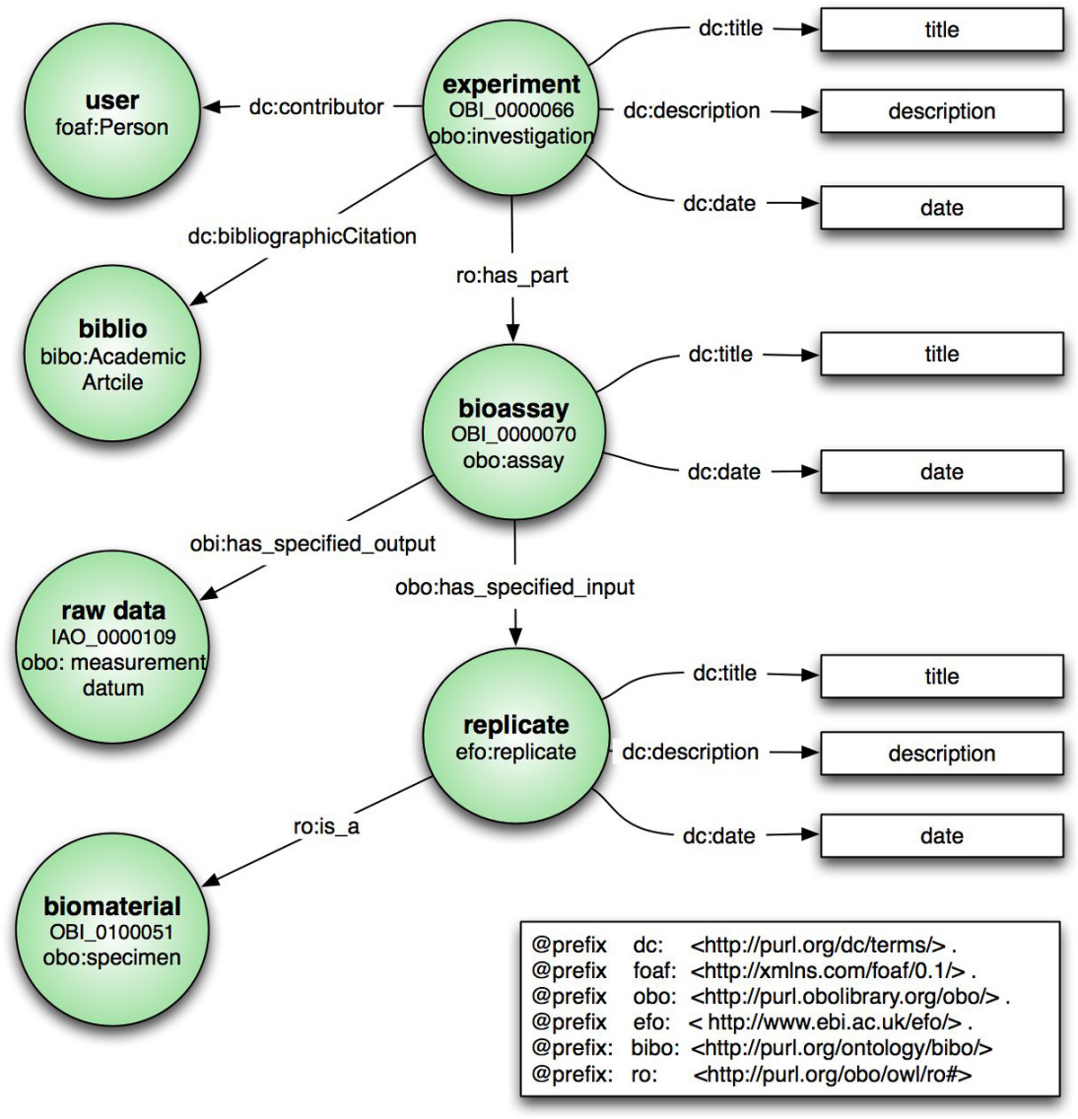
**Data Model **. Data model outlining the relationship between the experiment, its assays and biomaterials. The Drupal content types are indicated as green circles with the mapping listed underneath. Arrows indicate the relationships.

The main content type within eXframe is an experiment. It describes the experiment and its meta-data including title, description, contributors, design, citations, and links to external resources such as GEO [28] and ArrayExpress [8]. The experiment content type is mapped to the OBI investigation class *obo:investigation*. The experiment's "publication" meta-data is represented using the Dublin Core ontology [29]. However, we are currently evaluating the PAV ontology [30] as it provides more detailed and precise provenance information. For example, the Dublin Core ontology specifies the relation *dc:date*; but does not provide precise information as to whether the date is the "submitted date", "published date" or "last updated date". The researchers that conducted the experiment are represented as Drupal users with a profile and mapped to *foaf:Person *in the FOAF ontology [31]. While we do not specify the principal investigator (for the sake of simplicity), one could use VIVO [32] to do so. Bibliographic citations are represented using the Drupal biblio module and mapped to the bibliographic ontology, BIBO [33]. These classes and mappings are illustrated in Figure 2.

The experiment class also describes the overall protocol; measurement type and includes the experimental-factors, which can be exploited by bioinformaticians for data analysis. Experiments are composed of assays represented by the bioassay content type. The bioassay content type is mapped to *obo:bioassay *and specifies the technology platform used and other assay details. Bioassays are typically performed on several replicates specified by the replicate content type and mapped to *efo:replicate *(OBI only models replicate design and analysis). Each replicate is associated with the biological material on which the assay is conducted and is specified by the biomaterial content type. Thus technical replicates reference the same biomaterial, whereas biological replicates reference the unique materials used for the assay. The assays have raw data as their output. Data transformations and analyses conducted on the raw data are currently not represented, but are included in future plans for the system.

**Figure 3:**
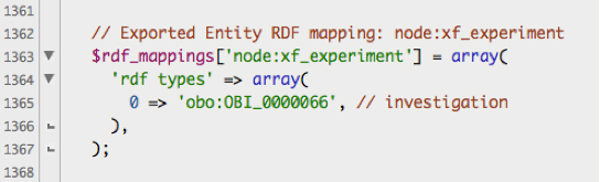
**Ontology mapping code**. Excerpt from exframe.entity_rdf.inc showing how Drupal classes are mapped to external ontologies.

Biomaterial is deeply annotated using Drupal Taxonomies and mapped to various controlled vocabularies and ontologies. In the eXframe default package, the organism, tissue type, cell type, disease state and chemical treatment taxonomies are mapped to NCBI Taxonomy (NCBITaxon) [34], FMA [12], CL [11], Disease Ontology (DO) [13] and Chemical Entities of Biological Interest Ontology (ChEBI) [35] terms, respectively. EFO [10], NCI Thesaurus [36] or Breda Tissue Ontology (BTO) [37] is also used to increase coverage when required. Biomaterial properties and their mappings are configurable and can be easily customized to a particular domain as required. The mappings of the main content types (experiments, bioassay, citation, biomaterials etc.) to ontologies are configured in PHP code, in a single file (an excerpt of which is shown in Figure 3). Attributes of the experiment, bioassays, and biomaterials that can be defined via structured vocabularies are stored as Drupal taxonomies. For example, "Cell Type", an attribute of the biomaterial, is represented as taxonomy. Each term in the taxonomy is mapped to a class or classes in external ontologies. Thus, "Fibroblast" a term in the "Cell Type" taxonomy, is easily added, edited and mapped to ontologies through the web interface.

### Linked data & SPARQL endpoint

We use the Drupal RDF modules to produce RDF using the mappings discussed above. RDF generated using the Drupal modules [26] is indexed into an RDF store powered by the ARC2 PHP library [38]. A SPARQL endpoint is also published by this RDF store. The RDF indexer in Drupal is designed to be backend-agnostic and allow for any RDF store to be plugged in. We're using ARC2, which is sufficient for our needs, but other stores can be used depending on the size of the dataset, or particular SPARQL features that might be needed.

Some of the data in the repository is kept private until the researchers publish their work. To maintain privacy, we utilize two stores: one of which solely contains the public data, and whose SPARQL endpoint is publicly available; the other which contains the entire data and is kept secure using an API key. The secure, administrative endpoint is used by R scripts (described in the next section) to access data for query and analysis by members who have access authorization. The other benefit of having decoupled stores is that we have the flexibility of optimizing the performance and scalability of each store independently from the other.

### R Integration

We wanted to provide programmatic access to the repository data to retrieve experimental information in a manner that is independent of the Drupal database schema. The R statistical programming language [39] and platform is a popular tool for analyzing genomics data. Thus, we decided to provide support for accessing RDF data and the SPARQL endpoint using R. The publicly available R packages to access RDF data are not yet fully featured; for example the SPARQL package doesn't support DESCRIBE queries. Hence the RDF package that does support DESCRIBE statements was used to provide information about the resources. Using the package, first the experiment RDF is used to obtain information about the assays, and then the assays provide information about the biomaterial (See relationships in Figure 2). The RDF package also had problems; it is hindered by UTF8 encoding issues. The resulting R scripts included in the eXframe package produce data structures compatible for analysis with R packages such as BioConductor [40,41].

## Results

### Case study: Stem Cell Commons

Stem Cell Commons (SCC) is a project of the Harvard Stem Cell Institute (HSCI) to freely share biomedical data, tools and resources within the research community [42]. Our platform, eXframe, was first implemented independently for the Blood genomics program at HSCI, and then later extended to support all researchers at the Institute, as the repository of Stem Cell Commons. Data from both the previously developed Blood Genomics store and the Stem Cell Discovery Engine (SCDE) [43] was merged into the eXframe-based SCC database.

Genomics datasets are actively curated into the database; currently the repository contains over 200 datasets from 20 laboratories representing 4 organisms and 119 different cell types and 39 tissue types. Results based on approximately half of the datasets (86) have been published in scientific journals, and these datasets are therefore available to the public.

All bioassays and samples have been deeply annotated with ontologies. First we used the OBI ontology [9] for the main entities (experiment, biomaterial and assays) as described in the data model section. Dublin Core [29] and FOAF [31] were used for the metadata and researcher respectively. The ontologies used to annotate the biomaterials are listed in Table 1. All the Stem Cell Commons public data is available as Linked Data as well as a SPARQL endpoint as described in the next sections.

**Table 1:** Ontologies used in Stem Cell Commons.

Content Type	Attribute	Ontology
Biomaterial	Organism	NCBITaxon [34]

Biomaterial	Development Stage	EFO [10]

Biomaterial	Tissue Type	FMA [12], EFO [10], BTO [37]

Biomaterial	Cell Type	CL [11], EFO [10]

Biomaterial	Disease State	NCI Thesaurus [36]

Biomaterial	Treatment	CHEBI [35], NCI Thesaurus [36]

Following ontologies were used to annotate the samples (biomaterial content type).

### RDF generation

**Figure 4:**
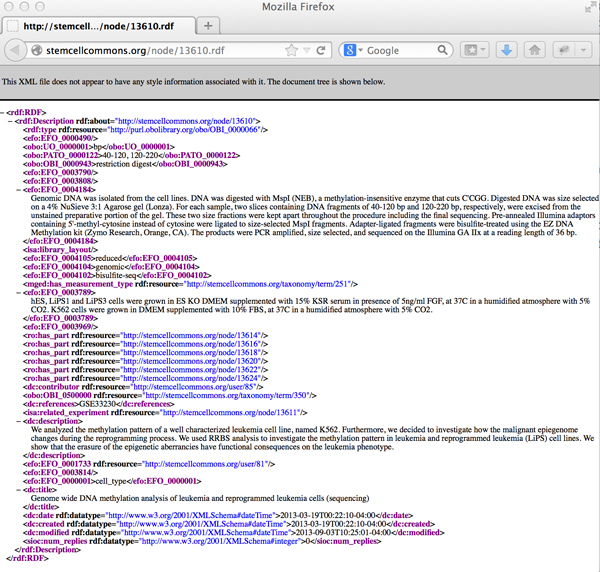
**Screenshot of experiment RDF**. Linked data from Stem Cell Commons illustrates use of DC, FOAF and OBI ontologies to describe an experiment, which is a DNA methylation experiment performed on various cell-lines with different passages. Available at: http://stemcellcommons.org/node/13610.rdf.

RDF for the experiment, bioassay and biomaterials are automatically generated using the Drupal RDF modules as described previously. A screenshot of actual RDF output for an experiment curated in the Stem Cell Commons is depicted in Figure 4. It is a next-generation sequencing experiment performed by a HSCI researcher and measures DNA methylation (using bisulphite sequencing) in the leukemia cell line K562, reprogrammed leukemia cell lines (LiPS) and the human embryonic stem cell line H1. From Figure 4, we see how the Dublin Core ontology provides the provenance information for the experiment. The bibliographic citations and external references are stated. The assay resources that are part of the experiment are listed using the *has_part *relation. The experiment has 6 assays performed on the cell lines with various passages. The protocol details are mostly described using a combination of OBI terms when available or EFO terms. The measurement type, an important attribute to identify which analysis tool to run, is described using the deprecated MGED ontology [44,45], as this term doesn't exist in any other ontology. The measurement type value - "DNA Methylation Profiling (Bisulphite Sequencing)" - is however described in OBI. The experimental factor (cell-line in this case) is also stated.

The DNA Methylation differences were measured in the various cell lines. The link to each of the biomaterials and corresponding RDF is available from the main experiment page (http://stemcellcommons.org/node/13610). Again, the biomaterial properties - organism, tissue, cell-lines and disease state properties were fully annotated using ontologies (details listed in Table 2). All terms were mapped to the normalized OBO Foundry ontologies [46] except "H1" where the EFO ontology was used. Such deep annotation with ontologies not only provides disambiguation; but also more importantly allows us to fully utilize the relations and properties that are defined in the external ontologies, as described in the next section. While annotation with ontologies providing term ratification is available in several repositories, SPARQL query capabilities like ours are not commonly available.

**Table 2:** Biomaterial Property Mappings.

Attributes	Value	Mapping
organism	Human	obo:NCBITaxon_9606

tissue	Blood	obo:UBERON_0000178

cell-line	K562	obo:CLO_0007060

cell-line	H1 (hESC)	efo:EFO_0003042

disease state	Myeloid Leukemia	obo:DOID_8692

obo:http://purl.obolibrary.org/obo/		

efo:http://www.ebi.ac.uk/efo/		

All biomaterial properties are mapped to OBO Foundry ontologies, or the EFO ontology.

### SPARQL query

**Figure 5:**
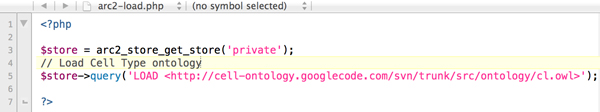
**Code snippet to load external ontologies**. The two lines of code are required to connect and load the CL ontology.

**Figure 6:**
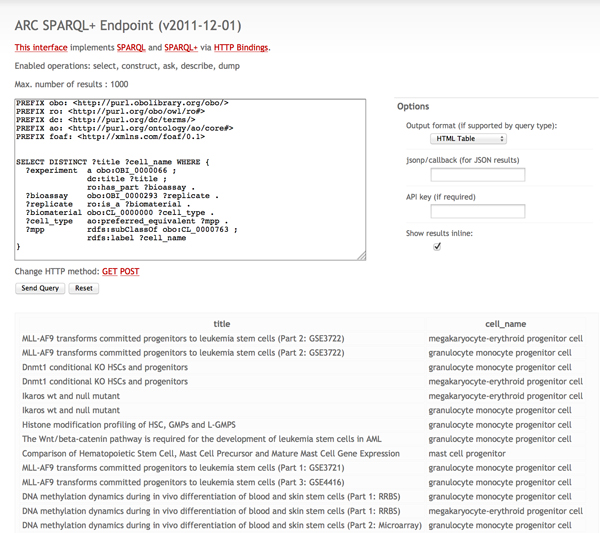
**SPARQL query run on Stem Cell Commons public endpoint**. Screenshot of SPARQL query run on the public Stem Cell Commons endpoint that integrates repository data with the CL ontology.

We list a query to find experiments done on mouse, hematopoietic stem cells in Table 3 that can be run on the SCC public SPARQL endpoint [47]. The public endpoint returns the 14 publicly available datasets whereas the admin endpoint can access all 25 records. We can load and integrate with external ontologies, such as the CL ontology, into the triple store using easy-to-use Drupal APIs to the ARC2 library [38] (see Figure 5). Then we leverage the properties and relationships defined in CL to find all the experiments performed on myeloid cells (CL_0000763) defined as "A cell of the monocyte, granulocyte, mast cell, megakaryocyte, or erythroid lineage." The query returns all available experiments performed on myeloid cells - granulocyte monocyte progenitor cell, megakaryocyte-erythroid progenitor cell, mast cell progenitor, myeloblast, monoblast, metamyelocyte, myelocyte and promyelocyte (Figure 6). Similar queries to find experiments on cells involved in a pathway or using synonyms defined in CL can also be performed.

**Table 3:** Sample SPARQL query.

PREFIX obo: <http://purl.obolibrary.org/obo/> PREFIX ro: <http://purl.org/obo/owl/ro#> PREFIX dc: <http://purl.org/dc/terms/> PREFIX ao: <http://purl.org/ontology/ao/core#> PREFIX foaf: <http://xmlns.com/foaf/0.1> SELECT DISTINCT ?title WHERE { ?experiment a obo:OBI_0000066 ; dc:title ?title ; ro:has_part ?bioassay . ?bioassay obo:OBI_0000293 ?replicate . ?replicate ro:is_a ?biomaterial . ?biomaterial obo:CL_0000000 ?cell_type . ?cell_type ao:preferred_equivalent obo:CL_0000037 . ?biomaterial obo:OBI_0100026 ?organism . ?organism ao:preferred_equivalent obo:NCBITaxon_10090 . }

Sample SPARQL query to retrieve experiments performed on mouse hematopoietic cells.

## Discussion

We have developed a reusable framework for creating genomics experiment knowledge bases with powerful human and machine interfaces including user-friendly GUI, R interface and SPARQL query against semantic experiment descriptors in RDF. Using the platform, researchers in academic or private institutions can manage their experiments and build genomics data repositories that are compliant with the Semantic Web standards. The structured repository serves as an institutional memory of research done in a laboratory and facilitates data publication. Not only does the eXframe platform make data sharing easy, it also allows researchers and the bioinformatics community to query this data via SPARQL in a flexible manner, while respecting data privacy. This was a major enhancement from the previous version. The new platform was deployed for the Stem Cell Commons project. In the results section, we demonstrate how to query the SCC data and the CL ontology in a single query, thus successfully exploiting the relationships stated in the CL ontology and integrating it with the repository information.

An important aspect of the work was to map the different elements of an experiment to and annotate bioassays and samples with existing biomedical ontologies. Our goal was to reuse rather than create yet another new ontology; but the approach had its challenges. To the extent possible, we use orthogonal ontologies as defined by the Open Biomedical Ontologies (OBO) foundry [46]. There was no single ontology that defined all the required classes and relationships; we had to use a heterogeneous mix of ontologies and each had to be individually maintained within our system. Often terms are missing or are not an exact match and a few times we had to use the deprecated MGED ontology (example presented in RDF generation section in Results). Another issue faced was the stability of resource identifiers. For example, the new version of the CL ontology includes identifiers (URIs) whose path is different from the old ones. While the old URIs resolve to the new ones, our databases and SPARQL endpoint had to be manually updated. Overcoming these challenges was a necessary step, as standardized representation of experiments is required for interoperability.

By creating a framework for new repositories that applies existing biomedical ontologies and publishes Semantic Web data, we not only lower the barrier to producing genomics experiment data compliant with the Semantic Web standards, but also provide a powerful mechanism to query data across knowledge bases from different domains. Although federated SPARQL queries are not supported by the RDF store we used, it is a first step towards interoperable genomics data. Given that eXframe was designed to allow any RDF store in the backend, federation could be achieved by choosing a different store with federation capabilities. As multiple research centers adopt eXframe, one can envision running queries across centers and with other biomedical knowledge bases; thus fully exploiting the power of the Semantic Web.

Querying and integration across databases is crucial to translational medicine where the need to bridge clinical and biological information is significant. To further enhance the integration capabilities, our next step will be to include the results of the computational analysis in the SPARQL endpoint. For example, this will allow us to query for gene expression changes in a pathway, spot histone modifications that result in expression changes, and identify transcripts whose expression is affected by transcription factor binding.

There are several databases that use ontologies to annotate the data; such as the ones listed in the Background section - Gemma repository [14], Chemical Effects in Biological Systems (CEBS) database [15] and Oncomine [16] ). The annotation is successfully utilized to make within-database queries. However flexible queries across knowledge resources cannot be done without the use of Semantic Web technologies such as those we provide. While the EBI Expression Atlas RDF platform provides powerful tools to query the public Array Express data; our reusable platform *enables institutions to create their own endpoint, and then query and integrate it with the vast web of existing knowledge bases*.

## Availability and requirements

eXframe is freely available at:

https://github.com/mindinformatics/exframe, under the GPL version 2 free software license. The eXframe framework runs on a LAMP stack, and uses the PHP and R programming languages. The web application is supported on all modern browsers.

## Competing interests

The authors declare that they have no competing interests.

## Authors' contributions

Emily Merrill and Stéphane Corlosquet were responsible for all software development. Sudeshna Das led the overall concept, architecture and project. Tim Clark and Paolo Ciccarese served as Semantic Web experts and Tim also advised on open-source collaborative software development. All authors contributed to the manuscript.

## Declaration

Authors declare that publishing charges were covered by funding from the Harvard Stem Cell Institute.

This article has been published as part of *Journal of Biomedical Semantics *Volume 5 Supplement 1, 2014: Proceedings of the Bio-Ontologies Special Interest Group 2013. The full contents of the supplement are available online at http://www.jbiomedsem.com/supplements/5/S1.
